# Supplementary material for: Quantification and Localisation of New Brain Lipid Synthesis Using Deuterium Oxide and High Resolution Mass Spectrometry
Source: Angew Chem Int Ed Engl. 2026 Mar 15;65(18):e24636. doi: 10.1002/anie.202524636 (PMC13110756; doi:10.1002/anie.202524636)
Supplement: Supplementary file 7 — Supporting File 7: anie71849‐sup‐0007‐SuppMat.docx [file ANIE-65-e24636-s007.docx]

**Experimental Section**

**Mice**

8-week-old male C57BL/6J mice were obtained from Australian BioResources (ABR, Mossvale, NSW) and kept under a 12 h light/dark cycle with *ad libitum* access to food and water. Mice were monitored at least twice weekly. Experiments followed the Australian Code of Practice for the Care and Use of Animals for Scientific Purposes and were approved by the University of Sydney animal ethics committee (project 2022/2133).

At 10 weeks of age, mice were randomly assigned to receive standard chow pellets (Ctrl, n=4), pellets containing 0.2% (w/w) cuprizone (Merck #C9012) for 5 weeks (Cpz, n=4), or cuprizone pellets for 5 weeks, followed by standard chow for 2 weeks (Rem, n=4). ^2^H_2_O was purchased from Merck (#151882), filter-sterilised, and provided at 25% (v/v) in the drinking water during the final 2 weeks of the diet. A fourth group received drinking water only while on chow (n=1), cuprizone (n=1), or chow following cuprizone (n=2).

**Brain Tissue Preparation**

Mice were anaesthetised via isofluorane inhalation, then transcardially perfused with 0.9% saline. Brains were collected and halved sagittally. Corpus callosum tissue was dissected from one hemisphere using a 1 mm dissecting matrix and stored at -80$^{\circ}$C. The other hemisphere was snap-frozen on dry ice. Sagittal sections of 15 µm thickness were prepared from the frozen hemisphere using a Shandon Cryotome FSE cryostat (Thermo Fisher Scientific), mounted onto Superfrost plus microscope slides (Thermo Fisher Scientific), and stored at -80$^{\circ}$C.

**Lipid Extraction**

The dissected corpus callosum tissue was homogenised in 500 µL of 20 mM N2-hydroxyethylpiperazine-N-2-ethane sulfonic acid (HEPES) buffer, pH 7.4, containing 10 mM KCl, 2 mM Na_3_VO_4_, 5 mM NaF, and complete protease inhibitor cocktail (Merck #11836170001), using a QSonica ultrasonication system at 4 °C, with 70% amplitude, 30 s on/off cycle for 10 min. The protein concentration of the homogenates was measured by bicinchoninic acid assay (Thermo Scientific #23225).

Lipids were extracted from 100 µL of corpus callosum homogenate using a two-phase methyl-tert-butyl-ether (MTBE)/methanol/water (10:3:2.5, v/v/v) protocol.^[1]^ Homogenates were mixed with 850 µL MTBE and 250 µL methanol containing internal standards: 30 nmoles β-Sitosterol; 5 nmoles each of PC 19:0/19:0, TG 17:0/17:0/17:0, and CE 17:0; 2 nmoles of SM 18:1;O2/17:0, GlcCer 18:1;O2/17:0, PS 17:0/17:0, PE 17:0/17:0, PG 17:0/17:0, PI 15:0/18:1[D7], and DG 15:0/18:1[D7]; 0.5 nmoles of SHexCer 18:1;O2/17:0, Cer 18:1;O2/17:0, LacCer 18:1;O2/12:0, LPC 17:0, LPE 17:1, and LPS 17:1 (Table S6, Supporting Information). Samples were sonicated for 30 min in a 4 °C water bath. Phase separation was induced by the addition of 112 µL Milli-Q water. Samples were vortexed and centrifuged at 2000 x g for 5 min, and the upper organic phase was collected in 5 mL glass tubes. The remaining aqueous phase underwent two additional extractions using 500 µL MTBE and 150 µL methanol followed by sonication for 15 min and phase separation with 125 µL water. Organic phases from the three extractions were combined and vacuum-dried overnight in a Savant SC210 SpeedVac (Thermo Fisher Scientific). Dried lipids were reconstituted in 200 µL of 25% high performance liquid chromatography (HPLC) grade methanol/25% 1-butanol/50% Milli-Q water containing 0.1% formic acid and 10 mM ammonium formate, centrifuged at 2000 x g for 10 min to pellet the insoluble material, and 150 µL was transferred to glass HPLC vials.

**Liquid Chromatography-Tandem Mass Spectrometry**

Lipidomic data was acquired using a Thermo Fisher Q Exactive HF-X Quadruple-Orbitrap mass spectrometer coupled to a Vanquish HPLC.^[1]^ Lipids were resolved on a Waters C18 Acquity UPLC column (2.1 × 100 mm, 1.7 μm pore size). The mobile phase flow rate was 0.28 mL/min using a 25 min binary gradient: 0 min, 80:20 A/B; 3 min, 80:20 A/B; 5.5 min, 55:45 A/B; 8 min, 35:65 A/B; 13 min, 15:85 A/B; 14 min, 0:100 A/B; 20 min, 0:100 A/B; 20.2 min: 80/20 A/B; 25 min: 80:20 A/B. Mobile phase A consisted of 10 mM ammonium formate, 0.1% formic acid in acetonitrile:water (60:40); mobile phase B consisted of 10 mM ammonium formate, 0.1% formic acid in isopropanol:acetonitrile (90:10). Data was acquired in full scan/data-dependent MS^2^ mode (resolution 60,000 FWHM at *m/z* 200, scan range 220–1600 *m/z*, AGC target 3e^6^, maximum integration time 50 ms) in both positive and negative mode for each sample. The ten most abundant ions in each cycle were subjected to MS^2^ using resolution 15,000 FWHM, isolation window 1.1 *m/z*, AGC target 1e^5^, collision energy 30 eV, maximum integration time 35 ms, dynamic exclusion window 8 s. An exclusion list of background ions was based on a solvent blank. An inclusion list of the [M + H]^+^, [M + NH_4_]^+^, and [M - H]^−^ ions for all internal standards was used. Positive mode data was not acquired for one sample from the remyelination group, due to a failed injection.

**Identification and Quantification of Deuterated Lipids**

Lipids from mice given normal drinking water (100% ^1^H_2_O) were annotated using LipidSearch v4.2 (Thermo Fisher Scientific), based on accurate precursor (5 ppm mass tolerance) and diagnostic product ions (8 ppm). A list of lipids, with their monoisotopic *m/z* and retention time, was generated (Table S1, Supporting Information). The predicted precursor *m/z* of the deuterated isotopologues was calculated by adding 1.0062767459 for each ^2^H atom (up to 20). Peaks for the non-deuterated monoisotope (M+0) and deuterated isotopologues (M+X, where X = number of deuterium atoms) in each sample were integrated using TraceFinder V5.1 (Thermo Fisher Scientific) based on precursor *m/z* (10 ppm window) and elution time (±0.1 min of the peak from the ^1^H_2_O control mice). IsoCor V2 (Python) was used to correct peak areas for naturally occurring heavy isotopes. The relative abundance of each isotopologue was calculated by dividing its peak area by the sum of all isotopologue peak areas (including the M+0 monoisotope) for that lipid. Deuteration was defined as having at least four adjacent isotopologues, with each isotopologue detected in at least three out of the four samples for any of the three treatment groups. Lipids were excluded from further analysis if the relative abundance of the M+0 monoisotope was <95% in ^1^H_2_O control mice (i.e. if apparent deuterated isotopologues comprised ≥5% of total peak area for that lipid in ^1^H_2_O control mice, indicative of interference).

Percentage deuteration was defined as the summed peak area of all deuterated isotopologues (M+1 – M+20) expressed as a percentage of the total peak area for that lipid (sum of M+0 – M+20). Absolute amounts of each isotopologue in each sample were estimated by dividing the peak area by that of the class-specific internal standard, and multiplying by the amount of internal standard added. Lipid levels were expressed relative to protein concentration of the homogenate (pmoles lipid/µg protein).

**Mass Spectrometry Imaging (MSI)**

Sagittal sections 2.0-2.2 mm lateral to the midline (Allen Mouse Brain Atlas levels 10-11) were used for MSI.^[2]^ Following removal from -80°C, tissue sections were dried for 15 min in a vacuum desiccator. MSI SPLASH mix (Avanti Polar Lipids, Birmingham, AL, USA, #330841) was prepared by diluting the stock solution 10 times with LC-MS grade MeOH and sprayed onto the sections using a HTX-TM Sprayer (HTX Technologies, Chapel Hill, NC, USA) as detailed previously,^[3]^ before finally coating in 20 mg of 2,5-dihydroxyacetophenone (Sigma-Aldrich, Castle Hill, NSW, Australia) matrix using an in-house built sublimation system. Sublimation was performed at 140 °C for 2.5 min. Samples were then recrystallised at 50^o^C using 1 mL of 0.5% ethanol for 90 s and stored in a vacuum desiccator until MSI.

MSI was performed using a prototype timsTOF Pro mass spectrometer (Bruker Daltonics, Bremen, Germany). To enable MSI, the system includes an atmospheric pressure matrix-assisted laser/desorption ionisation (MALDI) ion source that is combined with an inline dielectric barrier discharge plasma ionisation system (SICRIT, Plasmion GmbH, Augsburg, Germany) as detailed previously.^[4]^ MSI was performed in positive ion mode at a pixel size of 30 × 30 µm^2^ using beam scanning dimensions of 15 × 15 µm^2^. The laser was manually focused onto the sample prior to MSI and operated at a repetition rate of 5 kHz with 200 laser shots accumulated at each position. Desorbed species were collected in the heated inlet capillary (middle temperature of 360°C) and transferred to the SICRIT device for plasma post-ionisation which was operated at 1,500 V amplitude and 15,000 Hz frequency.

All data were processed using SCiLS Lab 2026a (14.00.17781, Bruker Daltonics, Bremen, Germany). The luxol fast blue images were manually annotated in QuPath 0.6.0 to define regions of interest,^[5]^ which were imported using the SCiLS Lab annotation plugin for QuPath. The average peak area at selected *m/z* values (± 15 ppm interval width, centroided mass errors typically less than 5 ppm) were normalized to their respective class-specific internal standard and annotated regions were exported as a CSV file for visualisation and statistical analysis using R Studio version 4.2. Given the mass resolution of the instrument’s time-of-flight (TOF) mass analyser (~35,000-40,000 FWHM @ *m/z* 750) and the absence of a pre-separation technique prior to mass spectrometry analysis, many mass peaks contain unresolved isobaric signals originating from other lipid species and their isotopologues. Examples include M+2 isotopes of lipid ions containing deuterium and/or ^13^C that are unresolved from monoisotopic lipids containing one less double bond (i.e type II isobaric interference), as well as deuterated lipid ions that may overlap with isotope clusters of other lipids. As such, the discussed lipids were selected based on inspection for minimal isobaric interference and spatial correlation between isotopologues to minimise the effects of unresolved isobaric interferences on data analysis and interpretation. Ion images were produced by extracting the raw ion images for selected masses and normalising per pixel to the respective internal standard using the SCiLS API, Python 3.13 and NumPy 2.0.2, and were converted into CSV files for visualisation using FIJI.^[6]^

**Luxol Fast Blue Staining**

Tissue sections were stained with luxol fast blue (LFB) and cresyl violet after MSI. The slides were washed in 100% methanol for 30 seconds to remove matrix, after which the tissue was rehydrated using 95% then 70% aqueous ethanol, followed by Milli-Q water for 2 min each. Sections were fixed in 10% neutral buffered formalin (Sigma-Aldrich, #HT501128) for 10 min and left to air-dry for 30 min inside a fume hood. Slides were placed in 0.3% HCl (prepared from 37% HCl, Sigma-Aldrich, #258148) in absolute ethanol for 3 min, rinsed for 3 min in 95% ethanol, and immersed in 1 mg/mL Luxol fast blue in ethanol (Sigma-Aldrich, #S3382) for 16 h at 58°C. Sections were allowed to equilibrate to room temperature and excess LFB washed with 95% ethanol, followed by a brief rinse in Milli-Q water. Slides were then immersed in a 0.05% lithium carbonate solution (Sigma-Aldrich, #255823) for 20 seconds to differentiate the sections followed by rapid, successive washes in 70% ethanol and Milli-Q water. This was performed for up to 1 min, until white matter areas were well-defined in blue against a clear grey matter background, and slides were then placed in Milli-Q water. The sections were counterstained with 1 mg/mL cresyl violet acetate solution (Sigma-Aldrich, #C5042) at 50°C for 20 min, then given a quick rinse in distilled water and differentiated in 95% ethanol for 3 min. Slides then underwent two changes of 100% ethanol for 2 min each and were cleared in xylene. Slides were cover slipped using DPX mountant (Sigma-Aldrich, #06522), dried overnight, and imaged with an Olympus Slideview VS200 in brightfield mode at 40x magnification.

**Statistical Analysis**

Statistical analyses were conducted using R Studio version 4.2. Lipids showing deuteration in two out of the three experimental groups were analysed by two sample independent t-test. Lipids showing deuteration in all three experimental groups were analysed by one-way ANOVA, followed by Tukey’s multiple comparisons test. p-values from both tests were binned and corrected for false discovery rate using the Benjamini-Hochberg test.

**Data Availability**

The datasets supporting the conclusions of this manuscript are available in Tables S1-S5. The LC-MS/MS raw data files are available at Metabolomics Workbench (DOI: 10.21228/M8655C). The MSI raw data files are available at Zenodo (DOI: 10.5281/zenodo.17504677).

**References**

[1] O. C. Marian, J. D. Teo, J. Y. Lee, H. Song, J. B. Kwok, R. Landin-Romero, G. Halliday & A. S. Don, ”Disrupted myelin lipid metabolism differentiates frontotemporal dementia caused by GRN and C9orf72 gene mutations” *Acta Neuropathol. Commun.* **2023**, *11*, 52.

[2] Q. Wang, S. L. Ding, Y. Li, J. Royall, D. Feng, P. Lesnar, N. Graddis, M. Naeemi, B. Facer, A. Ho, T. Dolbeare, B. Blanchard, N. Dee, W. Wakeman, K. E. Hirokawa, A. Szafer, S. M. Sunkin, S. W. Oh, A. Bernard, J. W. Phillips, M. Hawrylycz, C. Koch, H. Zeng, J. A. Harris & L. Ng, ”The Allen Mouse Brain Common Coordinate Framework: A 3D Reference Atlas” *Cell*. **2020**, *181*, 936-953.e920.

[3] M. Vandenbosch, S. M. Mutuku, M. J. Q. Mantas, N. H. Patterson, T. Hallmark, M. Claesen, R. M. A. Heeren, N. G. Hatcher, N. Verbeeck, K. Ekroos & S. R. Ellis, ”Toward Omics-Scale Quantitative Mass Spectrometry Imaging of Lipids in Brain Tissue Using a Multiclass Internal Standard Mixture” *Anal. Chem.* **2023**, *95*, 18719-18730.

[4] J. A. Michael, S. M. Mutuku, B. Ucur, T. Sarretto, A. T. Maccarone, M. Niehaus, A. J. Trevitt & S. R. Ellis, ”Mass Spectrometry Imaging of Lipids Using MALDI Coupled with Plasma-Based Post-Ionization on a Trapped Ion Mobility Mass Spectrometer” *Anal. Chem.* **2022**, *94*, 17494-17503.

[5] P. Bankhead, M. B. Loughrey, J. A. Fernández, Y. Dombrowski, D. G. McArt, P. D. Dunne, S. McQuaid, R. T. Gray, L. J. Murray, H. G. Coleman, J. A. James, M. Salto-Tellez & P. W. Hamilton, ”QuPath: Open source software for digital pathology image analysis” *Sci. Rep.* **2017**, *7*, 16878.

[6] J. Schindelin, I. Arganda-Carreras, E. Frise, V. Kaynig, M. Longair, T. Pietzsch, S. Preibisch, C. Rueden, S. Saalfeld, B. Schmid, J.-Y. Tinevez, D. J. White, V. Hartenstein, K. Eliceiri, P. Tomancak & A. Cardona, ”Fiji: an open-source platform for biological-image analysis” *Nat. Methods*. **2012**, *9*, 676-682.
